# Supplementary material for: Uncemented Tibial Fixation Has Comparable Prognostic Outcomes and Safety Versus Cemented Fixation in Cruciate-Retaining Total Knee Arthroplasty: A Meta-Analysis of Randomized Controlled Trials
Source: J Clin Med. 2023 Mar 1;12(5):1961. doi: 10.3390/jcm12051961 (PMC10003978; doi:10.3390/jcm12051961)
Supplement: Supplementary file 1 [file jcm-12-01961-s001.zip › jcm-2196942-supplementary.pdf]

Supplementary Materials

To analyze the reporting bias of each result, funnel plots were created using RevMan 5, as shown in Figures S1–12.

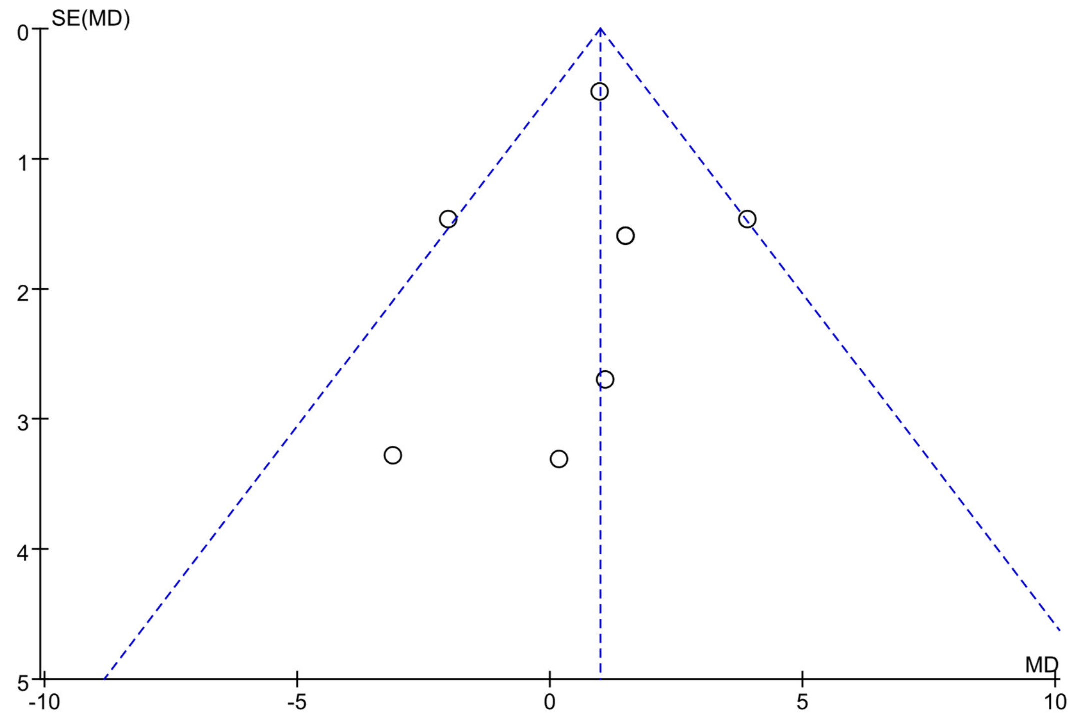

Figure S1. Funnel plot of KSKS.

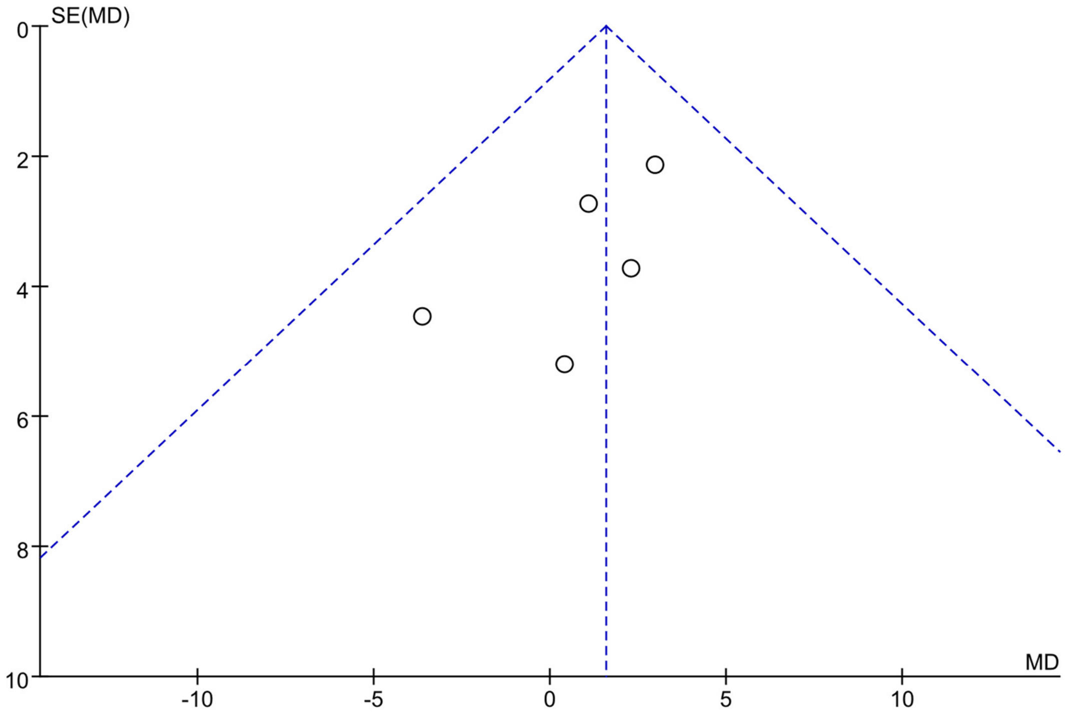

Figure S2. Funnel plot of KSFS.

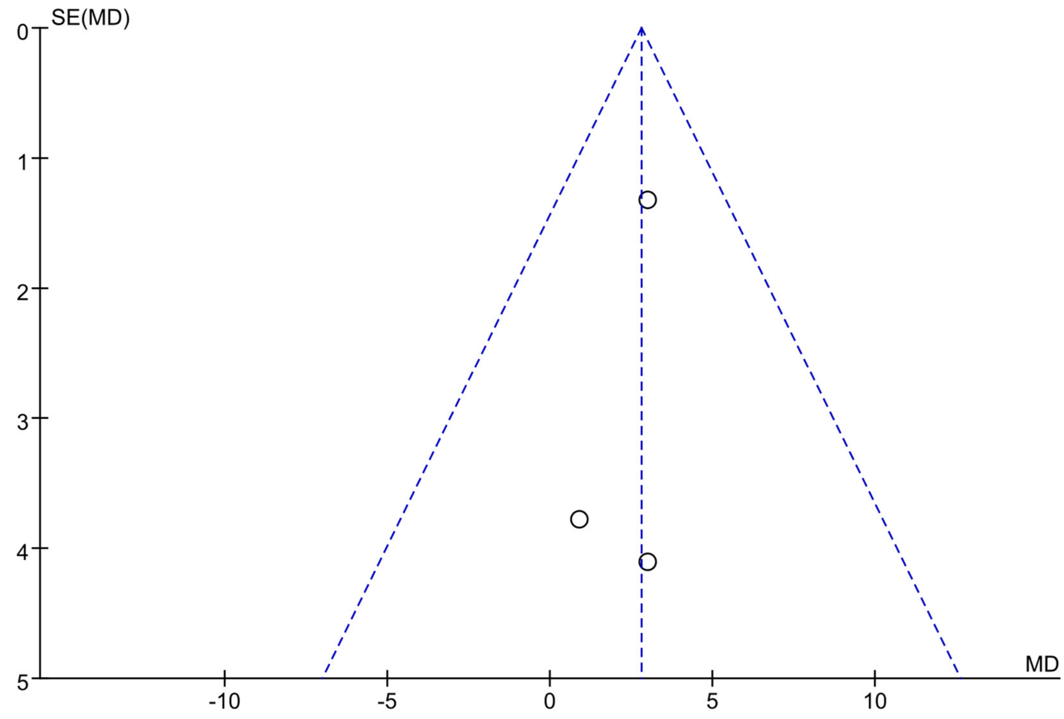

Figure S3. Funnel plot of KSS Pain.

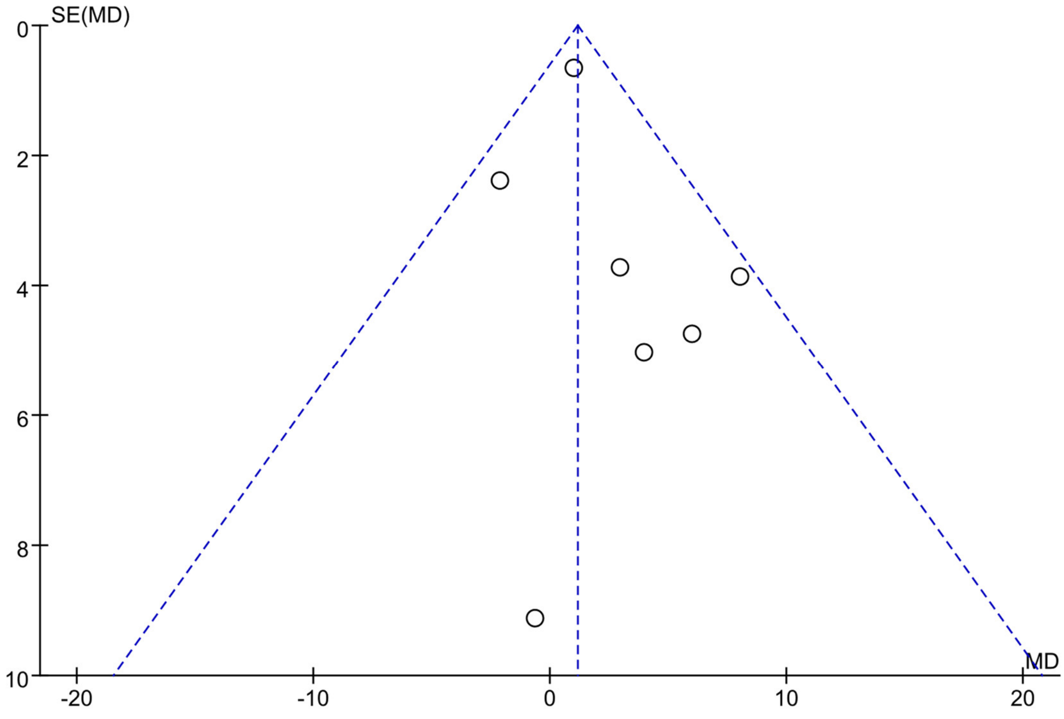

Figure S4. Funnel plot of ROM.

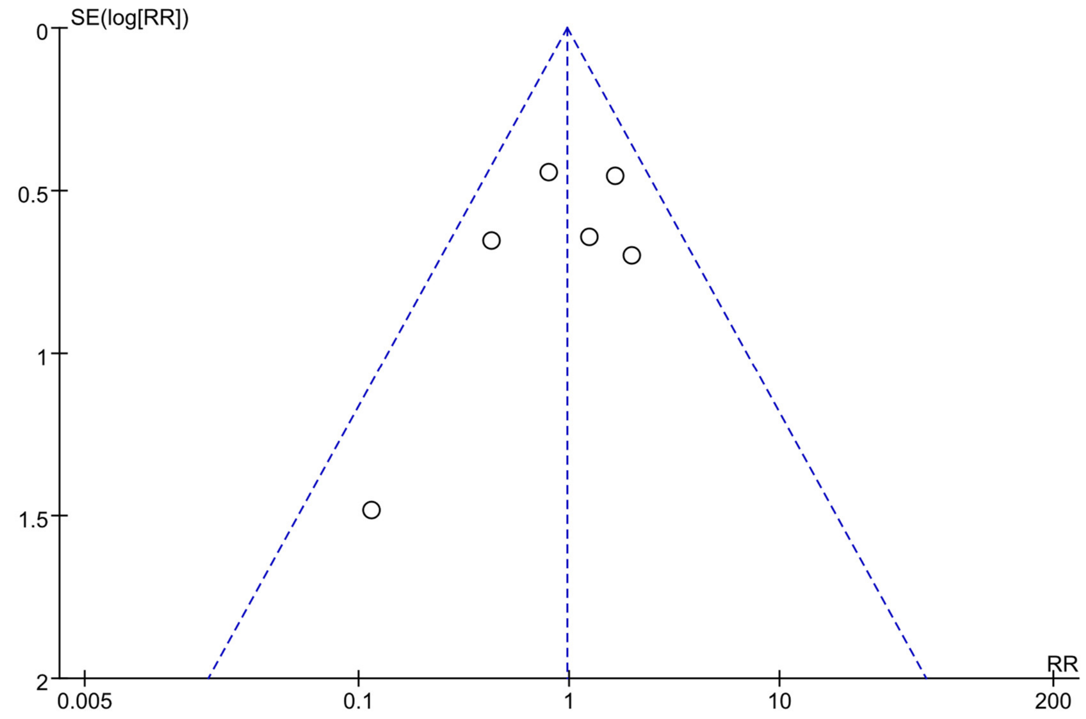

Figure S5. Funnel plot of RLL.

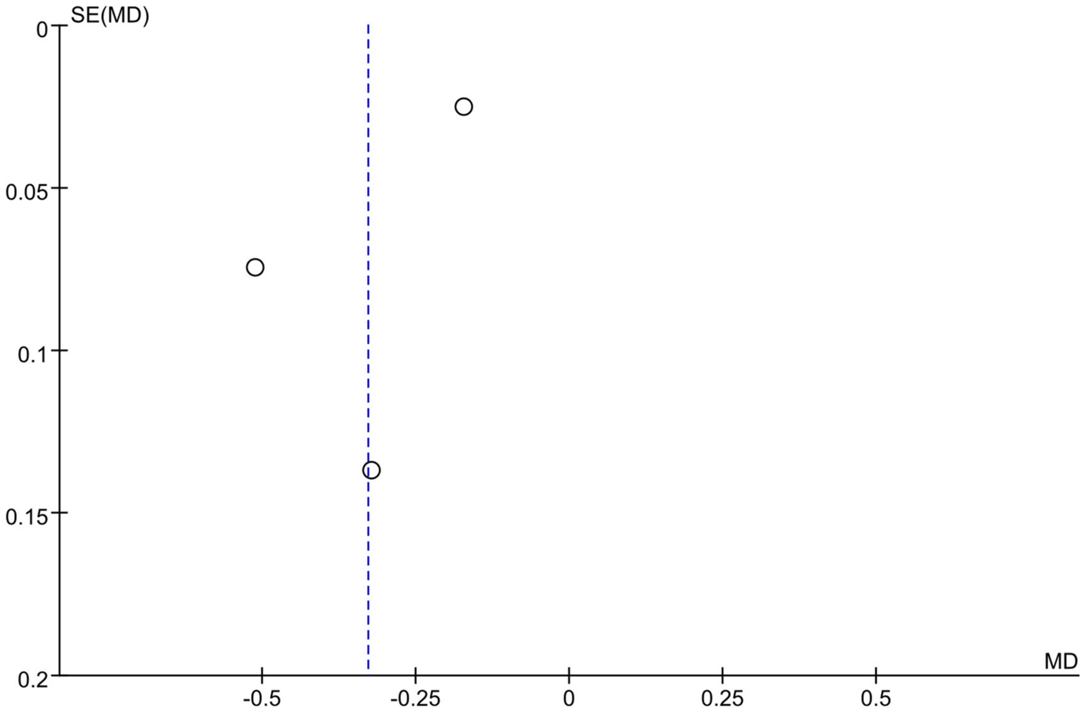

Figure S6. Funnel plot of MTPM.

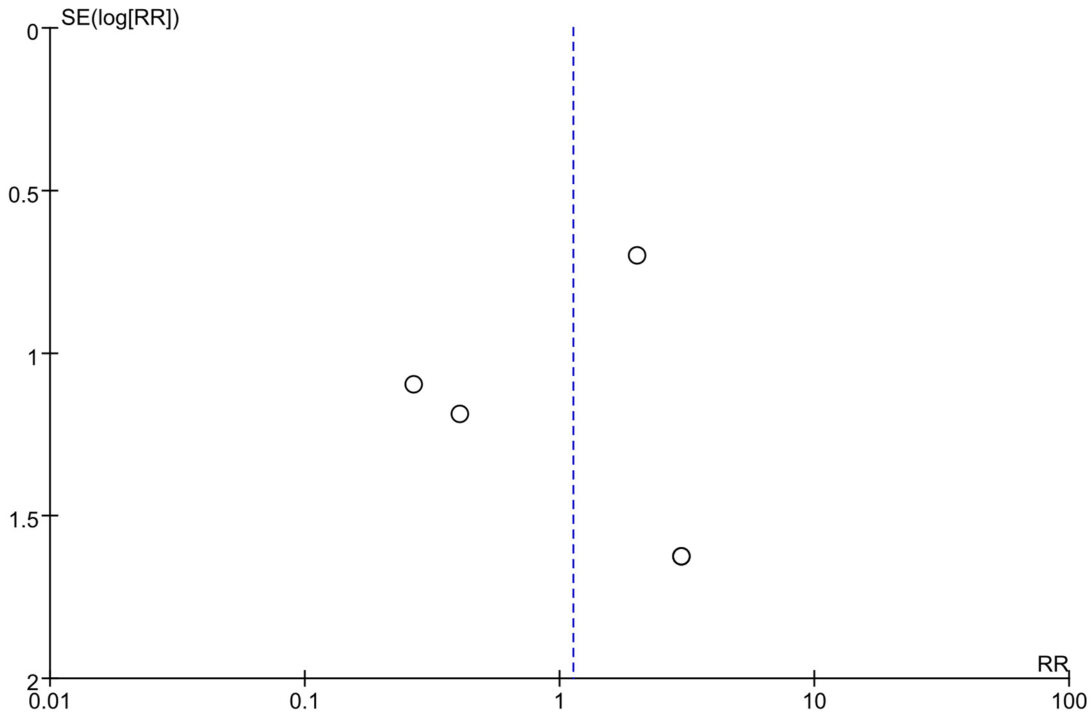

Figure S7. Funnel plot of aseptic loosening.

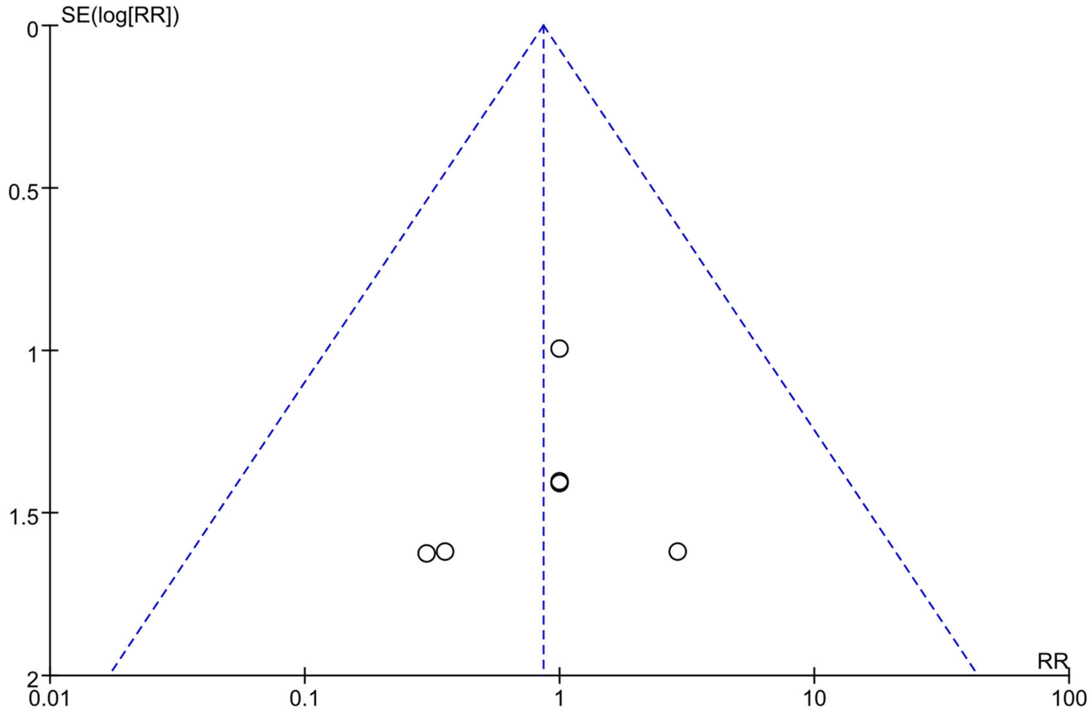

Figure S8. Funnel plot of infections.

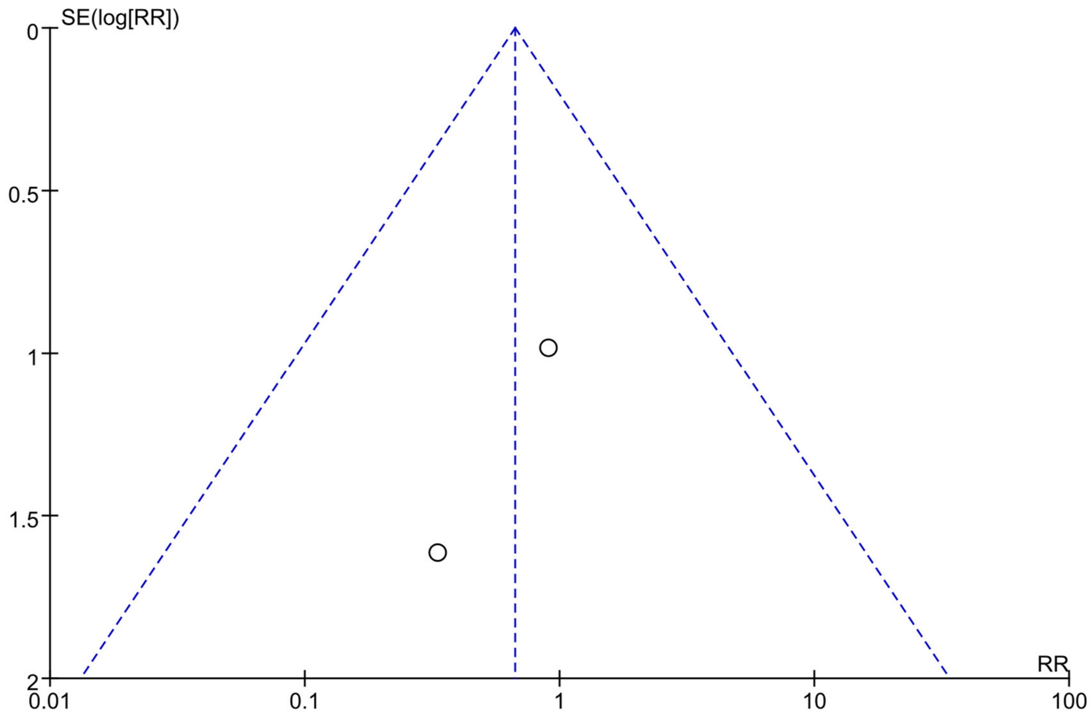

Figure S9. Funnel plot of thrombosis.

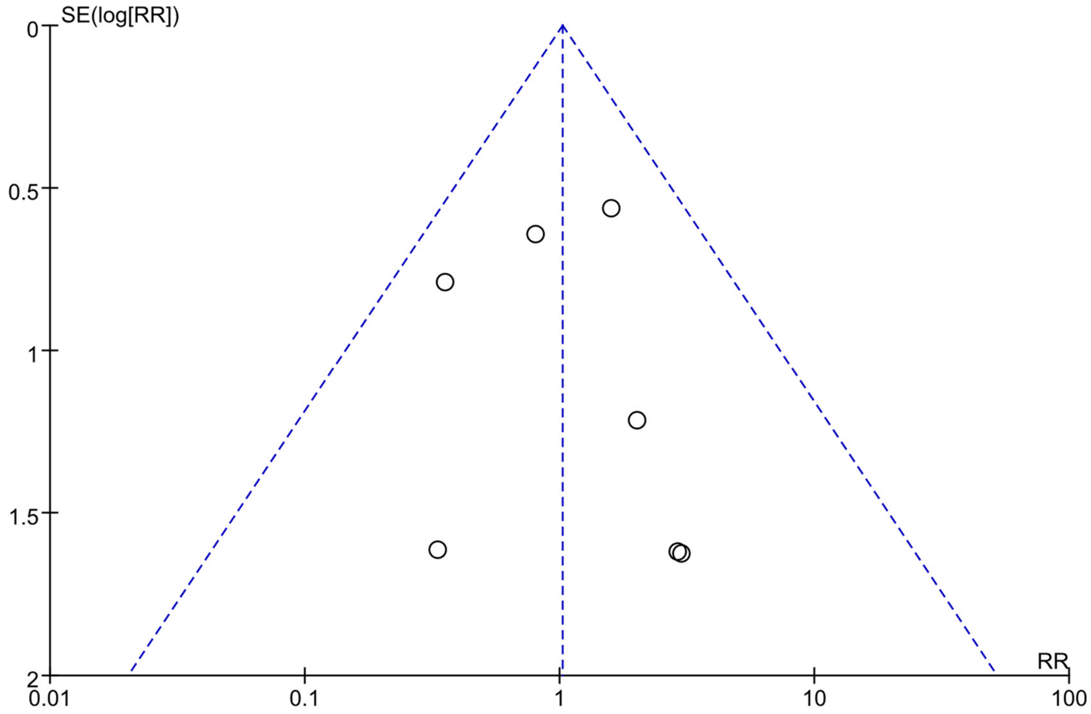

Figure S10. Funnel plot of revsions (without age limit).

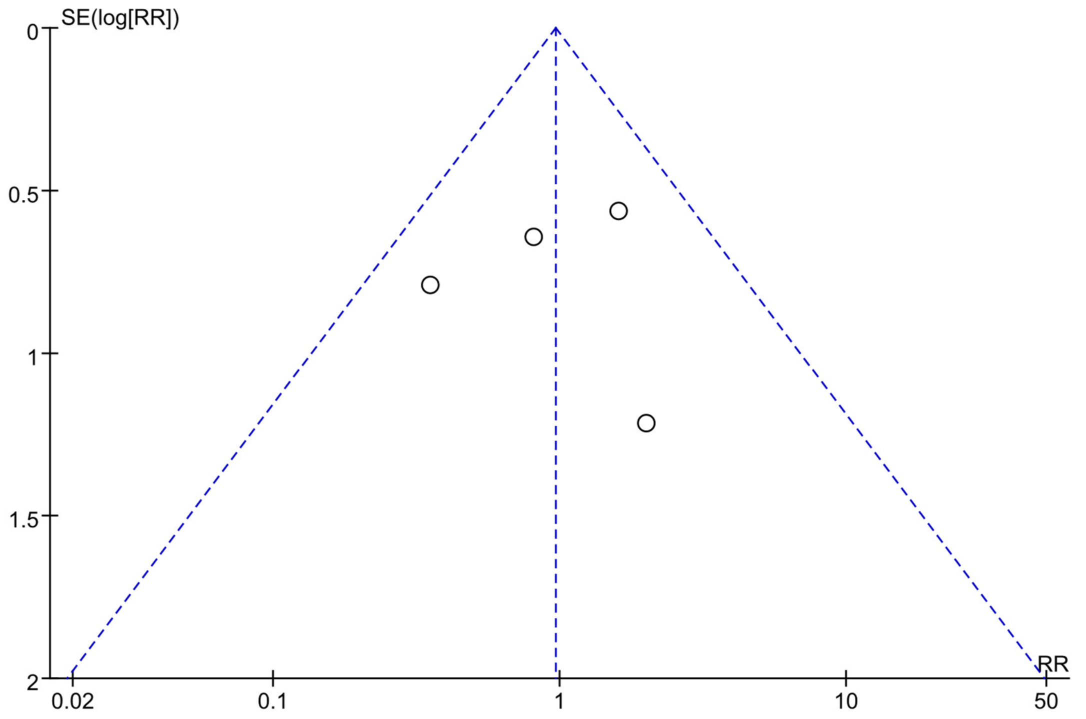

Figure S11. Funnel plot of revisions in young patients.

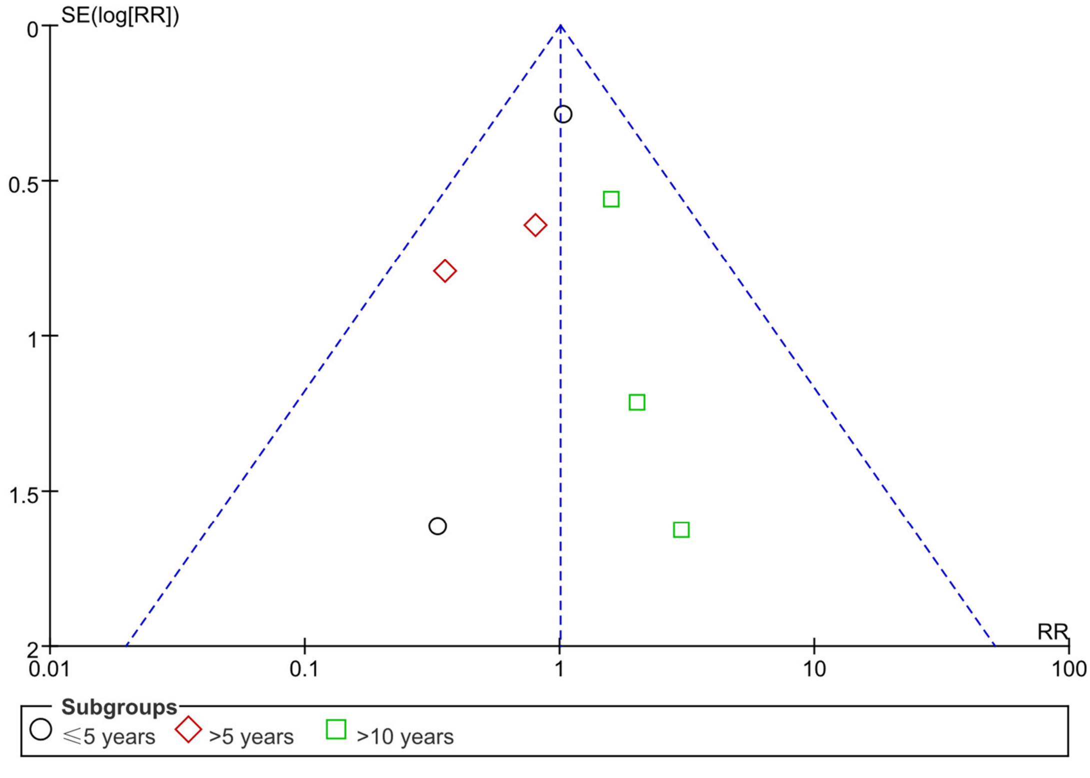

Figure S12. Funnel plot of revisions by time.
